# Learnings from a prison‐based drug treatment program on planning for release: A qualitative study

**DOI:** 10.1111/dar.13730

**Published:** 2023-08-13

**Authors:** Bebe D’Souza, Tony Butler, Anthony Shakeshaft, Ivan Calder, Katherine Conigrave, Michael Doyle

**Affiliations:** ^1^ Sydney Medical School, Faculty of Medicine and Health The University of Sydney Sydney Australia; ^2^ School of Public Health and Community Medicine, Faculty of Medicine UNSW Sydney Sydney Australia; ^3^ NHMRC Centre of Research Excellence in Indigenous Health and Alcohol, National Drug and Alcohol Research Centre, UNSW Sydney Sydney Australia; ^4^ Health through Walls New York USA; ^5^ Drug Health Services, Royal Prince Alfred Hospital Sydney Australia; ^6^ NHMRC Centre of Research Excellence in Indigenous Health and Alcohol, Central Clinical School, Faculty of Medicine and Health The University of Sydney Sydney Australia; ^7^ Edith Collins Centre (Translational Research in Alcohol Drugs and Toxicology), Drug Health Services Sydney Australia; ^8^ NHMRC Centre of Research Excellence in Indigenous Health and Alcohol, Sydney Medical School, Faculty of Medicine and Health The University of Sydney Sydney Australia

**Keywords:** alcohol, group treatment, illicit drugs, prison, rehabilitation

## Abstract

**Introduction:**

Prison‐based drug and alcohol group treatment programs operate in all Australian jurisdictions. With more than two‐thirds of people in prison having a history of substance use prior to incarceration, such programs are needed. There have been few published papers on the impact of attending group treatment programs in Australian prisons, and the research published to date has been predominately quantitative. We aim to report the experiences of males in prison who completed and those who did not complete a group‐based drug and alcohol program, to gain insight into their strategies for reducing harm from drug and alcohol post‐release.

**Methods:**

Qualitative thematic analysis of in‐depth interviews with 12 males who completed or were about to complete and 10 males who discontinued a prison‐based group drug and alcohol treatment program.

**Results:**

Program completers were more likely to have well‐developed plans to reduce drug and alcohol harms and maintain abstinence upon return to the community, which included creating healthier social networks. They also showed stronger insights into the factors that led to offending. Those who did not complete the drug and alcohol program appeared to rely on self‐will as the main way to reduce drug and alcohol harms, with less awareness of options for support services to reduce or stop drug and alcohol use.

**Discussion and Conclusions:**

Prison‐based drug and alcohol program engagement imparted useful information for program completers. Controlled trials are needed to examine whether such differences equate to improved outcomes after release.

## 1 INTRODUCTION

Drug and alcohol (D&A) use has long been identified as a contributing factor to criminal justice system engagement. Substance use disorder is common among people in prison worldwide [1]. It is defined by Diagnostic and Statistical Manual of Mental Disorders, Fifth Edition as when an individual's substance use meets 2 of 11 possible criterion which cover physical dependence, risky use, social problems and impaired control [2].

In Australia, a cross‐sectional survey of people in prison in the state of New South Wales (NSW) found that 16.5% self‐reported having used heroin on a daily or almost daily basis when last in the community [3]. Additionally, almost two in four of the same NSW sample reported using amphetamine‐type stimulants daily or almost daily when in the community [3]. There is a high rate of return to custody in NSW and Australia generally, with about half of the people released from prison returning to prison within 2 years of release [4]. Given people with substance use disorders are particularly vulnerable to reoffending [5], treating substance use in prisons has been suggested as one strategy to lower rates of recidivism [6, 7].

In NSW, on entry to prison there is a clinical health assessment by nursing staff which is often followed by brief motivational interviewing. A separate part of the intake is an assessment of behavioural issues that relate to criminogenic risks, that is, those factors which increase the likelihood of offending behaviour and are therefore appropriate targets for rehabilitation [8]. This assessment is done by therapeutic program staff and usually forms the referral pathway into D&A treatment programs [9, 10].

There are four broad categories of behavioural group treatment programs available in NSW and in other Australian prisons [11, 12, 13]. Cognitive behaviour therapy involves drawing links between thoughts and actions to challenge behaviour. There are a variety of cognitive behaviour therapy programs, which vary in structure and length [14]. Psychoeducation approaches, which are often akin to health promotion, provide educational session/s with participants so they can better understand the harms from D&A use, usually over short timeframes and with limited interaction time between staff and attendees [15]. Thirdly, abstinence‐based 12 Step programs such as Alcoholics Anonymous and Narcotics Anonymous are run in some prisons, with group meetings often facilitated by outside community volunteers who also provide peer support to prevent relapse [16].

The fourth type of program are prison‐based therapeutic communities and residential programs, with participants living separately from the general prison population [12]. These combine a range of strategies including individual counselling, cognitive behaviour therapy and psychoeducation, with programs usually lasting 3 months or longer. Therapeutic communities and residential programs have been identified as the type of prison‐based program most likely to reduce substance use post‐prison release [12, 17]. Although prison‐based D&A group treatment programs are available in every state and territory in Australia [13], few publicly available reports on program effectiveness are available [12, 18, 19]. Moreover, there are limited accounts of the experience of these programs in participants' own words [12].

This qualitative paper seeks to contribute to the evolving discourse on D&A group treatment programs in prison. We report on different perspectives between males in prison who attended and those who discontinued an in‐prison group D&A program. Our research took place at the Intensive Drug and Alcohol Treatment Program (IDATP) which is a residential program within a NSW Prison. We aim to gain a better understanding of factors that influence engagement and so to contribute to future D&A program designs. We explore whether a group program may change participants' perspectives on their D&A use, including planning for abstinence post release. To the best of our knowledge this paper is the first Australian qualitative paper on this subject.

## 2 METHODS

This qualitative study reports on 22 in‐depth voluntary interviews with males in an urban privately‐run prison in Sydney, NSW, who completed, did not complete or who were within 4 weeks of completion of an intensive D&A treatment program (IDATP) [18]. Thirty‐one males were first interviewed by co‐author Doyle, prior to them commencing the six‐month IDATP. They were then approached for re‐interview by Doyle 8–9 months later while still in custody. The intake interviews established a baseline of AOD use and prior treatment. The follow up interviews, which formed the basis of the paper, were undertaken to understand learnings from the program and the experience of the program for participants.

### 2.1 Participants

At follow‐up, six of the 31 participants had been released and were lost to follow‐up. Of the 25 men in prison, two refused to be re‐interviewed (one who continued in the program and one who had discontinued). One was willing to be re‐interviewed but could not be included in follow‐up interviews because he had not undertaken any part of the program.

Of the 22 interviewed, one participant, ‘Tom’, had left the program due to having been found by Corrective Services in possession of drug paraphernalia. Upon transfer, Tom commenced an alternative D&A three‐month program at the other facility. Because Tom remained active in a D&A behavioural treatment program his data were included in the group who continued. Another participant, ‘Ryan’, was included in the group who continued, as he had essentially completed the IDATP at the time of interview, although he had been placed in solitary due to a disciplinary matter.

### 2.2 Drug and alcohol program

The IDATP is an initiative of the NSW Government for people with substance use disorder and was established in February 2012 at John Moroney Correctional Centre, Sydney NSW. It was designed to target males in prison assessed using the Level of Service Inventory—Revised framework as having medium‐to‐high risk of re‐offending and having considerable substance use issues. NSW Corrective Services defines considerable usage as that which causes the individual serious adjustment problems and which is assessed as contributing markedly to their offending [20]. Completion of IDAPT, and therapeutic programs generally, is looked upon favourably by parole boards when determining early release.

At the time of this study there were between 10 and 16 people per IDATP group, and the group had 3–4 sessions per week. The program focused on addressing impulsive behaviour, understanding triggers for substance use and developing healthy behaviours. Admission to IDATP requires people to have enough time to complete the six‐month program, with greater than 12 months until earliest possible release date. Other criteria were low or medium security classification, no serious cognitive impairment, no acute mental health problems, and no serious misconduct in prison [18]. In addition to admission into IDAPT, study criteria included willingness and capacity to provide informed consent.

### 2.3 Interviews

Interviews for those who continued in the D&A program predominantly took place in the low security section of the prison where the D&A program was located. Interviews for those who had discontinued and had moved to another prison were on the phone or face‐to‐face, or in a different section of the same prison. All interviews were one‐to‐one with co‐author Doyle, who reintroduced himself as an Aboriginal man undertaking a PhD and reminded them of the study and reconfirmed their consent to participate.

The interviews were semi‐structured, between 30 and 45 minutes in length and centred on four topics: (i) D&A treatment; (ii) education and employment; (iii) prison environment; and (iv) reintegration into the community. The discussion was free flowing. Interviews were taped and professionally transcribed. Extensive field notes were taken on body language and tone.

### 2.4 Data analysis

A thematic analysis of the 22 follow‐up interviews was conducted by first author D'Souza with guidance from Doyle. A description of the methods and results relating to the first interviews have been published elsewhere [21, 22, 23].

Analysis was undertaken following the method used by Vaismoradi et al. and in four stages; initialisation to become familiar with the data, construction codes from the data, rectification of themes engaging with the literature and finalisation to develop a ‘story line’ from the data [24]. Co‐author Doyle set up an initial coding framework based on his previous work with the data [21, 22]. Doyle then coded two interviews, revised the framework and added definitions for the codes. D'Souza coded the 22 interviews (including recoding of the two coded by Doyle), adding new codes as needed. D'Souza and Doyle met regularly through the coding process to discuss and revise the codes and develop the themes. For our analysis, participants were separated into two groups, 12 who completed or were continuing a D&A program (the ‘continued’ group) and the 10 who had discontinued the program.

The NVivo software program was used for data management [25]. Identifying information such as names of localities have been omitted or altered to protect the confidentiality of the participants. Pseudonyms have been used for quotes below.

### 2.5 Ethics

Approval was granted to conduct the study from the Corrective Services NSW Ethics Committee and the Aboriginal Health and Medical Research Council of NSW Human Research Ethics Committee.

## 3 RESULTS

Demographic details of those who continued and those who discontinued are in Table 1. The groups were broadly similar in age and geographic location before incarceration. When employed, the majority of participants from both groups had worked sporadically as labourers. Observable differences between the groups included those who continued the program being more likely to have served more terms, with 75% being in their 4th prison term or more. In comparison, the majority (60%) of those who discontinued were completing their second sentence. The majority of those who continued the program identified heroin as their drug of choice (58%) whereas those who discontinued identified alcohol (40%), heroin (30%) or stimulants (30%).

**TABLE 1:** Participants in prison‐based intensive drug and alcohol treatment program.

	*Continued, N = 12*	*Discontinued, N = 10*
Location before prison		
Urban	4	7
Regional city	4	0
Regional town	2	3
Interstate	1	0
Age, years		
18–24	1	2
25–29	4	2
30–35	5	2
36–42	2	4
Highest formal education level		
Primary school	2	1
Early secondary	6	4
Mid secondary	4	5
Late secondary	0	0
Prison term		
First	1	1
Second	0	6
Third	2	3
4th+	9	0
Employment		
None	3	1
Labourer	2	2
Labourer (sporadically)	7	5
Office work	0	2
Preferred substance		
Heroin	7	3
Stimulants	4	3
Cannabis	2^a^	1
Alcohol	1	4^b^

^a^ Two participants who identified cannabis also listed other drug(s) in their preferences (amphetamine, alcohol) and so are counted more than once.

^b^ One participant in the preferred alcohol column also listed cocaine as a preferred drug and so is counted both for alcohol and for ‘stimulants’.

### 3.1 Program engagement

Self‐reported reasons for discontinuing were conflict with staff; moving prisons; voluntary withdrawal; being found with drug paraphernalia; and returning positive urinalysis for illicit drug use and being removed from the program. Some people listed multiple reasons.

The men who continued with IDATP spoke about participating in the D&A program and appeared to have attended most, if not all, of the classes with the trained facilitators. To remain in the D&A program participants were required to remain drug free as evidenced by a weekly urinalysis. Three men reported returning positive urinalysis results on a couple of occasions. This was described in the past tense, suggesting this was only in the early stages of the program. The three men appeared to be very grateful to have been allowed to remain in the program.

### 3.2 Abstaining from drug use in prison

Contact with people newly entering prison (including remandees), who had recently been using drugs was suggested to create a difficult social environment in which to abstain from drug use.

**Kurt (continued)**
‘You've got all these people coming in here who are just not trying. Don’t wanna get off drugs. They’ve just come from the street and they’re still using.’

Although a common theme among all 22 participants was the use of drugs in prison, only those who continued at the D&A program, including Tom, volunteered how to avoid such use. The men who continued indicated that although they were in an environment where pressure to use drugs could at times be high, this could be advantageous in practising relapse prevention skills. Carl discusses this as follows:

**Carl (continued)**
‘There's gonna be a lot of pressure out there too … but in here there's pressure in here too but you’re, you’re learning to, to deal with it [drug use].’

This ‘learning to deal with it’ was seen as a vital skill for living in the community post prison release for some participants. Three participants who had previously used drugs in prison stopped after commencing the D&A program. For Adam, the program appeared to be a turning point:

**Adam (continued)**
‘I was bad in gaol, using, smoking a lot of pot and stuff. Dirty urines. But, yeah, since I've been here, I've, the whole program, since I've been doing it for nine months, I haven’t. I’ve given clean urines.’

As suggested by participants Carl and Adam, the D&A program helped develop resilience that could be drawn on when in the community.

### 3.3 Post‐release planning

Different themes emerged between the group that continued and the group that discontinued about the expectations of future D&A use. Overall, the men who continued demonstrated more detailed and pragmatic plans to avoid D&A use. There are three themes around avoiding D&A discussed by both groups: (i) creating healthier social networks; (ii) structured time; and (iii) criminal behaviour.

#### 3.3.1 Creating healthier social networks

Both groups discussed the difficulties in maintaining abstinence when surrounded by friends and associates who were still using D&A. However, the group that continued in the IDATP appeared to have more developed plans to avoid using despite peer pressure. The continued group evinced strategies that appeared previously contemplated by their detail.

Continued group—changing social networks. The continued group discussed strategies of avoiding old social networks in the community, by developing new and more positive networks, which would help them avoid D&A use. Dean expressed a desire to disconnect from his old social network:

**Dean**
‘If I go back to my mates, you know, or something [yeah] or see ’em and knock into ’em, I’ll probably go stupid and go back to my old ways again, you know.'

As a result, Dean planned to move away from his hometown following his release. His comments convey a degree of self‐reflexiveness and responsibility for choosing of social networks. This was like other comments such as Ryan's, who stated he would avoid ‘certain suburbs’ of the regional town he grew up in to avoid using upon release. For Ryan it would be hard to move from his hometown and this was the next best option. For some, reluctance to return to old social networks included perceived need to avoid family members.

**Adam**
‘I, I wanna stay away from my siblings as much as I can … because I don’t wanna go back to that lifestyle.'

Adam's story illustrates the difficult choices to be made to prevent re‐exposure to D&A use in the community. Ian spoke about increasing his methadone dose as a strategy to avoid cravings.

**Ian**
‘That can just sort of keep me out of that circle and away from the limelight of the peer pressure.'

Beyond just reducing the chemical craving, for Ian, the opioid substitution therapy would help remove him from the need to interact with peers who were using drugs, hence reducing peer pressure as well.

Discontinued—changing social networks. Three participants who discontinued appeared to be relocating upon release however, unlike those who continued, moving did not appear to be part of a strategy to reduce peer pressure. They spoke as if the geographic change would make the difference but did not reflect upon social or peer change being important (i.e., not hanging around people who use drugs).

**Neil**
‘*…* I’m going to live with my parents when I get out and that's in … [small town].'

He then went on to say:

**Neil**
‘I don’t know anyone in that town. You know, all my friends and stuff are in Sydney … so I think yeah, that'll be a hard one to answer [avoiding drug use].'

When quizzed on how he would resist use, Neil suggested self‐will to be his primary strategy:

**Neil**
‘If I’m gonna go home and you know, use drugs in the community, it's not because I have to or it's not because of the people around me … I think, in a way, that, that comes down to, you know, self‐will.'

The group who discontinued in the program had a resistant tone in response to questioning:

**Interviewer**
‘Do you think you'll go back and use cocaine again?’

**Luke**
‘I’ll do my best not to. Don’t want to.’

**Interviewer**
‘How will you do that?’

**Luke**
‘Just avoid the crowd.’

**Interviewer**
‘Do you think you will start using? What if someone came to your door?’

**Luke**
‘No. I could resist.’

Most of the participants who left the program had an apathetic tone when quizzed upon their post released plans. They generally did not describe specific actions to avoid drug use, had limited consideration of social pressure, and where they did describe strategies, they were vague with a focus on self‐will.

#### 3.3.2 Structured time

A common problem discussed by both groups was the difficulties of having large amounts of free time which would lead to boredom, particularly if unemployed. Adam's comments highlight the issue:

**Adam (continued)**
‘Like, when you get out and you, and you can’t get a job … Like bored as well, getting bored. … [Yeah] you wanna use drugs.’

There was a marked difference between the group who continued and those who discontinued in their approach to handling that ‘free’ time.

Continued—Structured time. Those who continued had more detailed plans and strategies to reduce the amount of unstructured time post release. These men listed activities to keep themselves busy. As discussed by Lee:

**Lee**
‘It's, you know … [about] dropping bad habits and taking up better ones, new habits, positive habits … and healthier habits.’

Lee continued:

**Lee**
‘Spending time with family and friends … just, just normal everyday stuff … walking the dog … those kinds of things … [before prison] my pastime was … anything but, you know.’

Lee went on to talk about other activities he could do to fill up the day, as he worried that spare time could increase the chances of relapse. Lee spoke at length of his plans to pursue a career as a D&A counsellor/support worker.

**Lee**
‘I reckon community services (TAFE certificate). If you do this one course you can branch off from that course and do drug and alcohol or mental illness.’

The longer Lee spoke the more specific details he provided about post‐release plans. He readily listed steps including finishing his school certificate, undertaking additional training in community services at TAFE (a Technical and Further Education facility) and finally, completing an advanced certificate in D&A. When asked if he had previous plans like this before commencing the D&A program Lee stated: ‘*I never, I never had these kinds of goals, no’*.

Some participants who continued in the program made plans well beyond leisure activities to structure their time. This included participation in support groups, volunteering at community organisations and completion of vocational courses and higher degrees at university.

Discontinued—Structured time. Those who discontinued did not have realistic nor detailed plans to reduce the amount of unstructured time post‐release. When asked on how he would fill his time Mark comments:

**Mark**
‘Maybe, if I got to finish the program, I would have … You know what I mean? I would have learnt a bit more. But they, [expletive], didn’t even give me a chance.’

Mark seemed to believe it was the responsibility of the program to provide him with ideas to structure his time, he did not view this as his own responsibility. Where strategies were provided, they lacked detail:

**Cole**
‘Well, it's better just not to do it you know … I just wanna try get into some probably volunteer work or something you know. Something, do something good … you know what I mean?’

Although the expression of these plans by Cole are positive and show prosocial tendencies, he is non‐committal and uses hedging terms rather than direct language. Additionally, Cole's tone also indicated some ambivalence. Similarly, in a somewhat ambivalent tone, Owen describes his plans post release:

**Owen**
‘I suppose it's good to be accountable … like surround myself with positive, like get into maybe church or church group or something like that. You know what I mean?’

These comments could represent a constructive plan for Owen, however his language is similarly non‐committal. Owen did not discuss that he had ever been to church before, nor did he indicate a specific parish to volunteer at. Both Cole and Owen at least mention plans for their time, others indicated when prompted that they ‘do not know’ and just that they would not use drugs.

#### 3.3.3 Criminal behaviour

Participants who completed the D&A program, unlike those who discontinued, appeared to have more insight into their criminal behaviour and the circumstances within which it arises.

Continued—Criminal behaviour. Completed group members, frequently expressed remorse for their offence. Participants who continued were able to describe in some detail the friendship networks, relationship stressors and difficult life events surrounding their offences. In relation to his break‐and‐enter, Dean shows contrition:

**Dean**
‘Just done something I shouldn’t have done that day. I went out and did a crime and stuffed up.’

Dean spoke of a group of men, older than him who would get him to steal on their behalf. He declares ‘now they're not my mates’. When quizzed whether he came to this position due to age Dean replied:

**Dean**
‘No, not only that. Now in the program …. We talk about it … and yeah.’

Sam, also appears to have reconsidered his relationships, he credits the program for forcing him to reflect:

**Sam**
‘They really wanted you to get deep into what was going on in your life and how people were, and how you were treated.’

Sam continued:

**Sam**
‘It was a good exercise … once you sit down and you start writing from the very beginning, once, once you get to the end and you look back your life becomes a lot clearer. Like you see more clearly defined stages and more clearly‐defined warning signs.’

Men who completed the D&A program had also developed an understanding of the emotional triggers contributing to offending. For some it was stressors associated with family life, including financial pressure, partners or traumatic life events such as the suicide of a parent. Sam discussed the difficult personal circumstances surrounding his offending behaviour:

**Sam**
‘I was angry at the world after, after my dad killed himself and, and I was angry at the world. And, and, in a way, like I didn’t care. I, l lost, I, I, I lost care for everything.’

Although participants described a multitude of difficult life events, for some the D&A program appeared to be the first forum where they had been able to open‐up and describe their emotional experiences. It appears that the structure of the D&A program may connect participants to their actions and their emotions in a way that they were not able to previously.

Discontinued—Criminal behaviour. Participants who discontinued the D&A program showed less insight into the offending behaviour. One man, Ben, assaulted his girlfriend's ex‐partner but when quizzed about his assault commented:

**Ben**
‘Well, I didn’t do the crime. That's the thing.’

Ben admitted to the offence but believed his behaviour was justified as it was to protect his girlfriend from her ex‐partner.

Similarly minimising his offending behaviour, Tony comments ‘just one stupid mistake that put me in here’. In fact, Tony had served several sentences but had not thought more broadly about why that was. One man, Luke, who had been charged with driving‐related offences stated he:

**Luke**
‘Doesn’t plan on doing crime.’

However, he later said:

**Luke**
‘Got no drivers licence and that could be my downfall.’

When asked if he planned to take public transport he continued:

**Luke**
‘I’ll try and do that.’

Luke, like many who discontinued, displayed short‐term thinking and, while he clearly knew what his ‘downfall was’, he did not rule out further offending.

### 3.4 Community‐based support services

If not raised spontaneously in the conversation, the men were asked specifically, where in the community they can get help to prevent or to stop using drugs. The responses differed between groups.

#### 3.4.1 Continued

The continued group indicated that they would actively find the help they needed. They also knew of either the available services in the local area that they were going to live in upon release or where they could start looking for help to find these services.

**Carl**
‘I’d ring Justice Health or AMS and ask if there's AA or NA, or a D&A case worker. You know, stuff like that. I’d look for the right people that could help me …’

#### 3.4.2 Discontinued

The responses from the discontinued group gave limited or no specific responses to where they could find support. For example, Dan had to be prompted by the interviewer:

**Interviewer**
‘Do you know any organisations or anywhere you can go if you did want some help?’

**Dan**
‘Yeah, I do. I've seen a, I’d just probably see a private counsellor.’

**Interviewer**
‘Oh okay. Any sort of other places you can think of?’

**Dan**
‘Not really. … I wouldn’t do anything, group work or anything like that.’

The discontinued group did not indicate a proactive approach to finding D&A support services.

**Interviewer**
‘In terms of places, where you could get help?’

**Ray**
‘Family.’

**Interviewer**
‘Can you think of any services in your area?’

**Ray**
‘I wouldn’t have a clue.’

**Interviewer**
‘You wouldn’t know like to ring, ring an organisation or anything?’

**Ray**
‘I wouldn’t even know one.’

### 3.5 Summary: Post‐release plans

The group that continued were more ready and willing to answer questions about post‐release plans. As well as conveying a greater sense of preparedness, the continued group demonstrated an understanding of the difficulties of abstinence. The discontinuers focused on why they left the IDAPT program and overall, they spent less time speaking about their post‐release plans. The discontinuers were more self‐reliant in their plans to maintain abstinence, and less aware of resources that could help them. Overall, the discontinuers were interested in conversation regarding release from prison, but their tone conveyed a sense of disinterest in discussing plans to address D&A use. This was different from the continued group who were very willing to discuss such plans.

## 4 DISCUSSION

The men who remained in the D&A program appeared to have a greater understanding of how to avoid D&A use within and outside of the prison environment. They displayed more complex planning to avoid D&A use in the community and a more considered understanding of their offending behaviour than those who discontinued the program. While there may have been differences between the two groups at entry to the program, the interviews suggest that the men could describe useful skills and insights as a result of program participation.

### 4.1 Drug and alcohol use

Participants discussed the drug use of other people in prison, which is not surprising as this phenomenon is well known [5]. Participants described having a ‘dirty’ urinalysis (indicating drug use) themselves and for two men, this appeared to be at the start of the program and they were allowed to continue. Others in the discontinued group indicated they were discharged because of such a result. It should be noted that program rules would mean being discharged for ongoing use. A prison‐based program with an automatic discharge, without leeway for therapeutic staff discretion, might be unhelpful. Discussing a positive urinalysis test and giving an inmate opportunity to reflect and stop using could be beneficial. The two men who continued were extremely grateful for such an opportunity and for them, as well as other inmates, it could be that overcoming drug use in prison might be an indicator of commitment and motivation toward future personal change.

Participants expressed concern about moderating their behaviour around remandees, who are individuals being held in custody pending court appearances. Remandees were described as having more problematic drug use and as creating a challenging environment that threatened program adherence. This is in keeping with the literature which suggests that for therapeutic communities to be most effective, participants should be separated from the general prison population [26]. When considered in light of Prochaska and Declemente's ‘stages of change’ framework it is intuitive that being in a cell with someone who is not yet up to the same stage of change could be detrimental to the person who wishes to move from ‘contemplation’ to ‘action’ [27]. As such, when possible, inmates in a D&A program should be housed separately from the general prison population.

### 4.2 Social networks

Members of the continued group had plans to disconnect from old social networks. The nature and quality of social relationships can both encourage and inhibit recovery. Avoidance of certain people and places is therefore associated with successful abstinence [28, 29]. Pettersen et al. conducted semi‐structured interviews with 18 individuals who remained abstinent for at least 5 years. A key finding was that while it is important to maintain positive relationships, it is equally important to engage self‐agency at protecting oneself against negative relationships [30]. The continued group frequently demonstrated limit‐setting intentions in deciding who to remain close to and who to cut out of their lives. They actively planned to disconnect from problematic relationships even when this was difficult—as with Adam who planned to avoid members of his family. It is this self‐agency that the group who discontinued the D&A program were lacking—instead, they seemed to be at best passively moving locations without expressing a desire to remould their support networks in favour of their abstinence.

### 4.3 Structured time

The participants who continued in the D&A program expressed more detailed plans for how they would structure their time post‐release. Marlatt described classical taxonomies of high‐risk situations that may trigger relapse for alcohol dependent patients, including boredom leading to relapse [31]. Unstructured time was described by Kowalczyk et al. as ‘time spent in behaviour with no strict schedule or urgent goal’. This controlled trial concerning the use of clonidine as an adjunct to buprenorphine, provided participants with a portable device that would prompt them at four random intervals a day to record their current level of craving and any activities they were engaging in presently. The authors found that engaging in responsibilities is related to positive treatment outcomes in individuals receiving standard opioid agonist therapy [32]. Although our study was qualitative, it complements the literature suggesting that good treatment outcomes arise from commitments and responsibilities [33, 34, 35]. Those who remained in the D&A program had thought more deeply about what activities they would engage in post‐release. These activities went beyond occupational planning to include hobbies and social outings. If the men who continued the program go on to action these behaviours, it will put them in better stead to withstand temptation to use drugs in the community than those with fewer plans.

### 4.4 Criminality: Emotional communication

The D&A program appeared to provide a novel forum where men were able to discuss their internal emotional state. The prison population has a high prevalence of emotional burden [32]. This is contributed to by the situation inmates are in and the difficult life circumstances leading to incarceration. Kupers has argued that toxic masculinity has a chilling effect on emotional discourse within the prison environment [36]. The men who continued in the D&A program developed tools to express their emotions even if this had been difficult for them in the past. In comparison, those who discontinued the program did not express their emotions and showed limited insight, at times boasting or ‘big noting’ their capacities. A study interviewing prison guards found that that people in prison often withheld emotions due to wanting to ‘show bravado’ [37]. This is concerning as the inability to identify or express emotions, termed alexithymia, has been linked to drug use, as well as being a possible moderator of the risk of violent offence recidivism [38]. It appears the D&A program may have assisted participants to develop their emotional communication through workshops and structured problem solving.

### 4.5 Criminality: Attitudes toward reoffending

Participants who remained in the D&A program could describe both the short‐term gains of crime, as well as the long‐term consequences. The dual‐systems theory in criminology suggests that criminal decision making is governed by two distinct and interrelated systems [39]. The first is impulsive and circumstantial, how an individual responds to the immediate situation [39]. Mamayek et al. describe the second as slow ‘based on the contemplation and rational consideration of the costs and benefits of an action, particularly the long‐term costs against a given action’ [39]. Although distinct, these systems are also interrelated; the slower more thoughtful second system acts to regulate the actions ‘suggested’ by the first. This slower, more considered, thinking appears to be consistent with the responses from those individuals who continued at the IDATP. The men who continued had developed thoughts about both short‐term and long‐term outcomes, demonstrating a different calculus to those who discontinued. It appears the program, which had a strong focus on these subjects, had an impact on the thinking of these men. This could have a flow‐on effect in reducing the likelihood of reoffending.

### 4.6 Community‐based support services

Knowing where to find help in the community is likely a protective factor against escalating usage. Barriers to medical care for addiction during re‐entry into the community include a lack of knowledge about services [40]. Furthermore, people who are on parole and have a community‐based court order might be reluctant to admit relapse and to seek help. Justice Health and other sections of NSW Health, as well as Aboriginal and other community‐based services, can provide support and help people avoid escalating D&A use [41, 42], which in turn could help reduce likelihood of criminal offending. It was quite telling that the continued group compared to the discontinued group knew where or how to find help and support. It also bodes well that the members of the continued group express an intention to proactively seek such services.

### 4.7 Limitations

This study has several limitations. Although we have found that prisoners' perspectives of D&A use appear different between those that ‘continued’ and those that ‘discontinued’ the D&A program, there could be influences on this result other than the IDATP. Follow‐up interviews were recorded for participants who had completed differing amounts of the program—some only had a couple of months' exposure while others had completed the program fully. Participants who completed the program had served more sentences and therefore may have a higher intrinsic motivation to change [9]. Motivation of participants more broadly may have influenced differences in learnings taken by each group. Unfortunately, we did not ask specific questions around motivation to change at the time of the initial interviews. We recognise perceived differences in learnings may also be due to participants taking on a program ‘narrative’ or using learned language. Learnings may not be therefore representative of each group's ability to change [43, 44]. The findings are based on stated plans of participants, as our research did not include post‐release interviews. This would be an interesting area for future research.

## 5 CONCLUSION

Participants who remained in this intensive in‐prison group D&A program expressed differing perspectives on substance use than their peers who discontinued the program. Those who continued had more strategies to avoid drug use in the community, such as avoiding old social networks, engaging in more structured activities, and practising abstinence while incarcerated. In comparison, those who discontinued the program were more reliant on ‘self‐will’ to maintain abstinence; they demonstrated limited insight into avoiding drug use in the community. Participants who continued also appeared to develop skills in communicating about their emotions and were more likely to reflect on triggers for their offending behaviour and D&A use than those who discontinued. Further research, including controlled trials are needed to examine to what extent differences in participants at the start of the program influenced continuing or discontinuing. Also, such trials could examine whether these differences between the two groups translate into improved outcomes after release.

## AUTHOR CONTRIBUTIONS

Michael Doyle, co‐designed the original study and collected the original interview data. Bebe D'Souza coded the interview data for the present study and in collaboration with Michael Doyle was responsible for the analysis and interpretation of data. Bebe D'Souza drafted the article with support from Michael Doyle and revised the manuscript for publication. Tony Butler, co‐designed the original study, supported data collection, reviewed, and commented on each draft of the manuscript. Anthony Shakeshaft, assisted with development of the study, reviewed and commented on each draft of the manuscript. Ivan Calder, co‐designed the original study, provided expert advice on the drug and alcohol program being studied, reviewed, and commented on manuscripts. Katherine Conigrave, reviewed and commented on manuscripts, provided expert advice on clinical aspects of AOD treatment.

## FUNDING INFORMATION

This work was supported by the National Health and Medical Research Council through an Investigator grant (APP1193617; for MD) as well as through the Centre of Research Excellence in Indigenous Health and Alcohol (APP1117198) and through a National Health and Medical Research Council Practitioner Fellowship (APP1123456; for KC).

## CONFLICT OF INTEREST STATEMENT

The authors declare no conflicts of interest.
